# Supplementary material for: Alternative stable states, nonlinear behavior, and predictability of microbiome dynamics
Source: Microbiome. 2023 Mar 29;11:63. doi: 10.1186/s40168-023-01474-5 (PMC10052866; doi:10.1186/s40168-023-01474-5)
Supplement: Supplementary file 2 — Additional file 1: Figure S1. Experimental setting and microbiome data formats. [file 40168_2023_1474_MOESM1_ESM.docx]

**
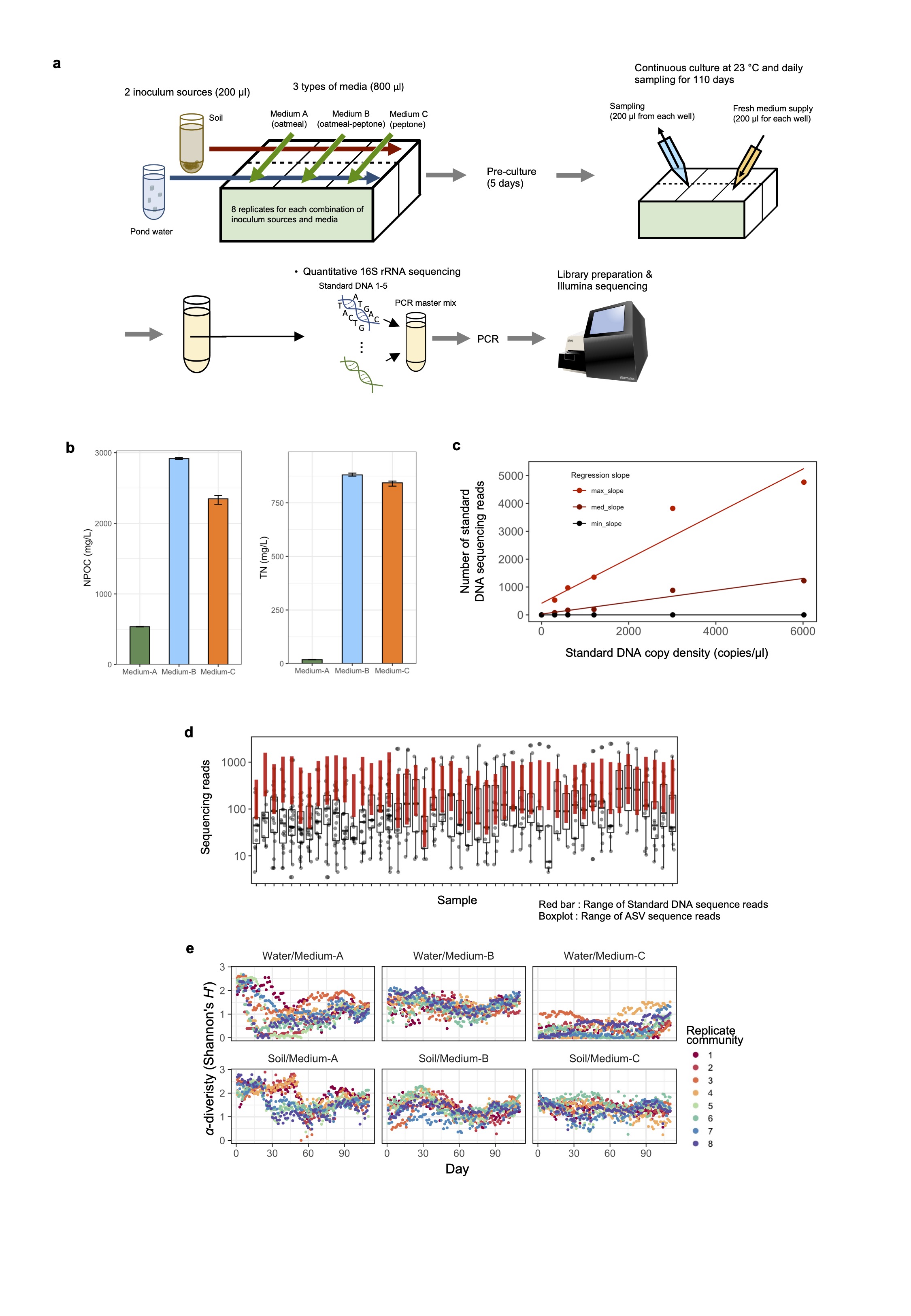
**

**Additional file 1: Fig. S1** Experimental setting and microbiome data formats. **a** Laboratory culture system. Source microbiomes from forest soil and pond water were respectively introduced into three types of media [Medium-A, 0.5% (w/v) milled oatmeal; Medium-B, 0.5% (w/v) milled oatmeal + 0.5% (w/v) peptone; Medium-C, 0.5% (w/v) peptone] with eight replicates. A fraction of the culture fluid was sampled every 24 hours and equivalent volume of fresh medium was added to the continual culture system throughout the 110-day experiment. After DNA extraction, five “standard DNA” variants with different concentrations were introduced into the amplicon sequencing analysis of the 16S rRNA region, yielding DNA copy number estimates of each prokaryote ASV in each replicate sample. **b** Concentrations of non-purgeable organic carbon (NPOC) and total nitrogen (TN) in each of the three types of fresh media. The bars represent ranges of triplicate measurements. **c** Example of calibration of 16S rRNA copy concentration. In most microbiome studies, only proportions of respective microbe’s sequencing reads to total sequencing reads (relative abundance; Additional file 3: Fig. S3) have been analyzed, while calibrated abundance information (absolute abundance; Additional file 2: Fig. S2) allows us to reconstruct population dynamics (i.e., increase/decrease through time-series) of respective ASVs in microbiomes. Five standard DNA sequences varying in concentration were added to PCR master mix solutions to infer relationship between DNA copy concentration and the number of sequencing reads in each sample. **d** Calibration of DNA copy concentration with the standard DNA gradients. The number of sequencing reads of each microbial ASV (boxplots and circles; black) was compared with that of standard DNA sequences (range of five standard DNA variants; red) in each sample. **e** *α*-diversity (Shannon’s *H′*) of ASVs through the time-series.
