# Supplementary material for: Alternative stable states, nonlinear behavior, and predictability of microbiome dynamics
Source: Microbiome. 2023 Mar 29;11:63. doi: 10.1186/s40168-023-01474-5 (PMC10052866; doi:10.1186/s40168-023-01474-5)
Supplement: Supplementary file 4 — Additional file 3: Figure S3. Dynamics of calibrated abundance. [file 40168_2023_1474_MOESM3_ESM.docx]

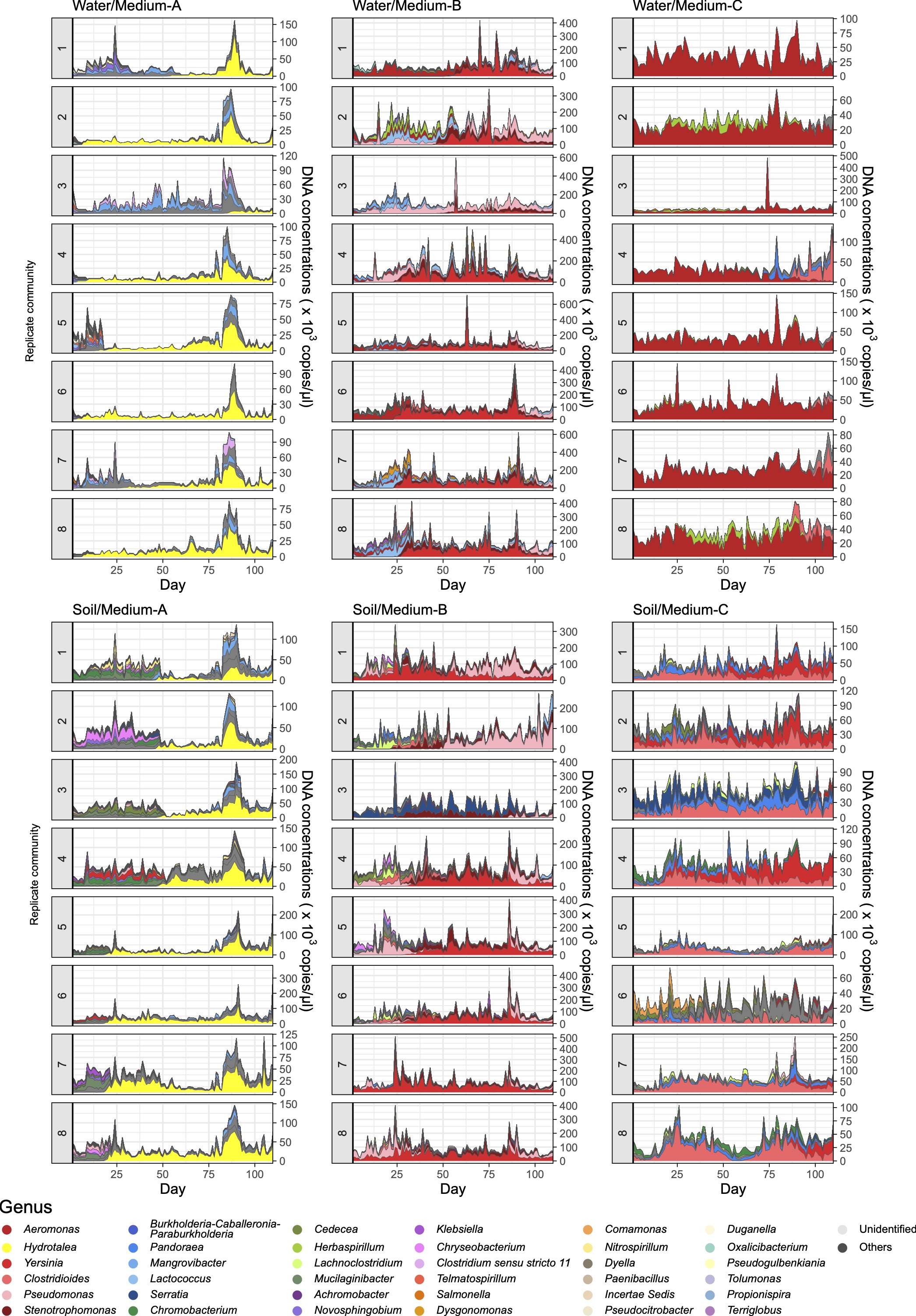


**Additional file 3: Fig. S3** Dynamics of calibrated abundance. For each replicate community of each experimental treatment, the changes of 16S rRNA gene copy concentrations (See Additional file 1: Fig. S1) are shown for each genus throughout the time-series. Note that each genus displayed in this figure can represent multiple microbial ASVs in the original dataset. Missing data points were interpolated as detailed in Methods.
