# Supplementary material for: Alternative stable states, nonlinear behavior, and predictability of microbiome dynamics
Source: Microbiome. 2023 Mar 29;11:63. doi: 10.1186/s40168-023-01474-5 (PMC10052866; doi:10.1186/s40168-023-01474-5)
Supplement: Supplementary file 5 — Additional file 4: Figure S4. Dynamics of relative abundance. [file 40168_2023_1474_MOESM4_ESM.docx]

**
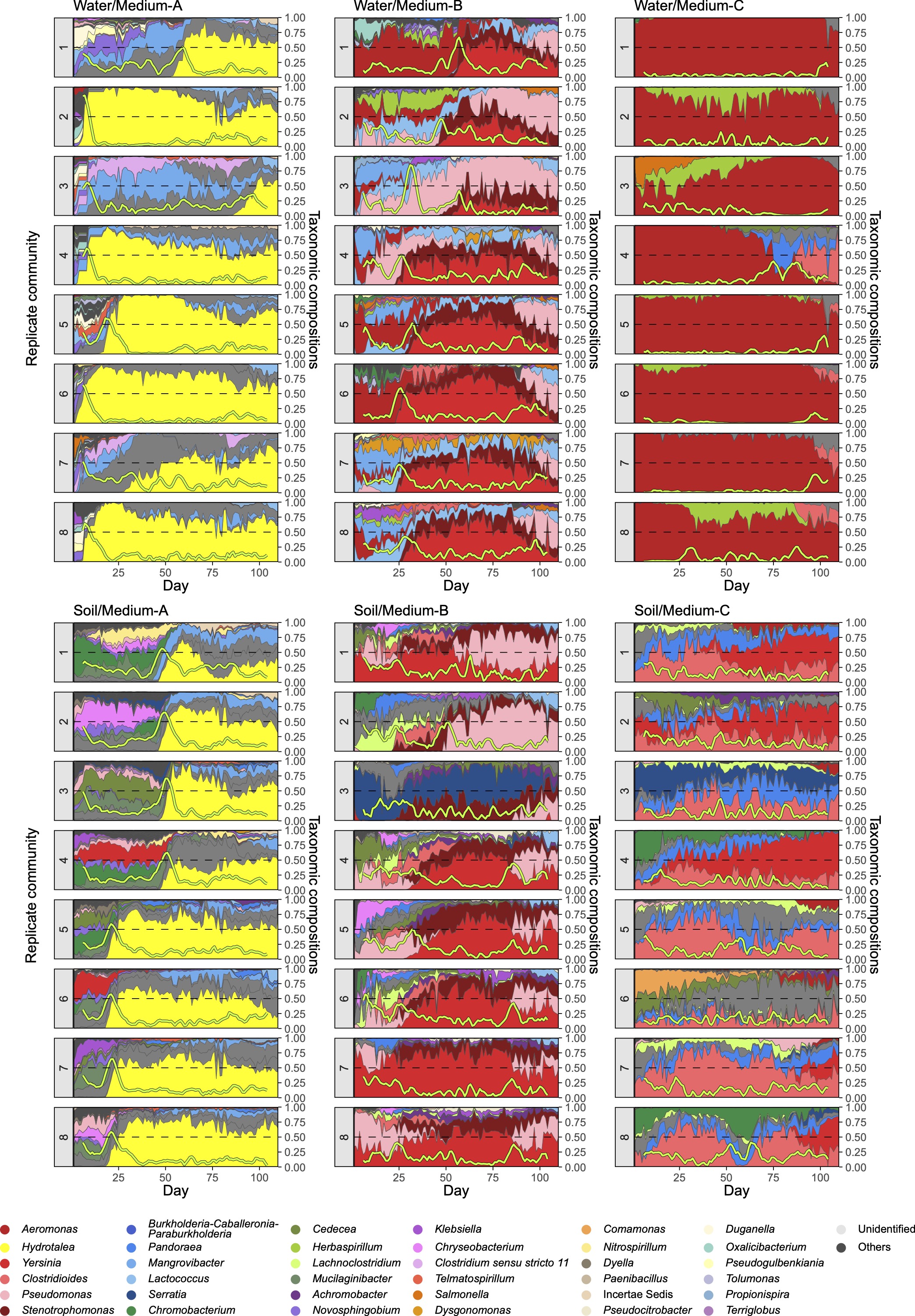
**

**Additional file 4: Fig. S4** Dynamics of relative abundance. For each replicate community of each experimental treatment, the changes of the relative abundance of the 16S rRNA region are shown for each genus throughout the time-series. Note that each genus displayed in this figure can represent multiple microbial ASVs in the original dataset. Horizontal line represents abruptness = 0.5. Missing data points were interpolated as detailed in Methods.
