# Supplementary material for: Alternative stable states, nonlinear behavior, and predictability of microbiome dynamics
Source: Microbiome. 2023 Mar 29;11:63. doi: 10.1186/s40168-023-01474-5 (PMC10052866; doi:10.1186/s40168-023-01474-5)
Supplement: Supplementary file 6 — Additional file 5: Figure S5. Distribution of stable states on the energy landscapes. [file 40168_2023_1474_MOESM5_ESM.docx]

**
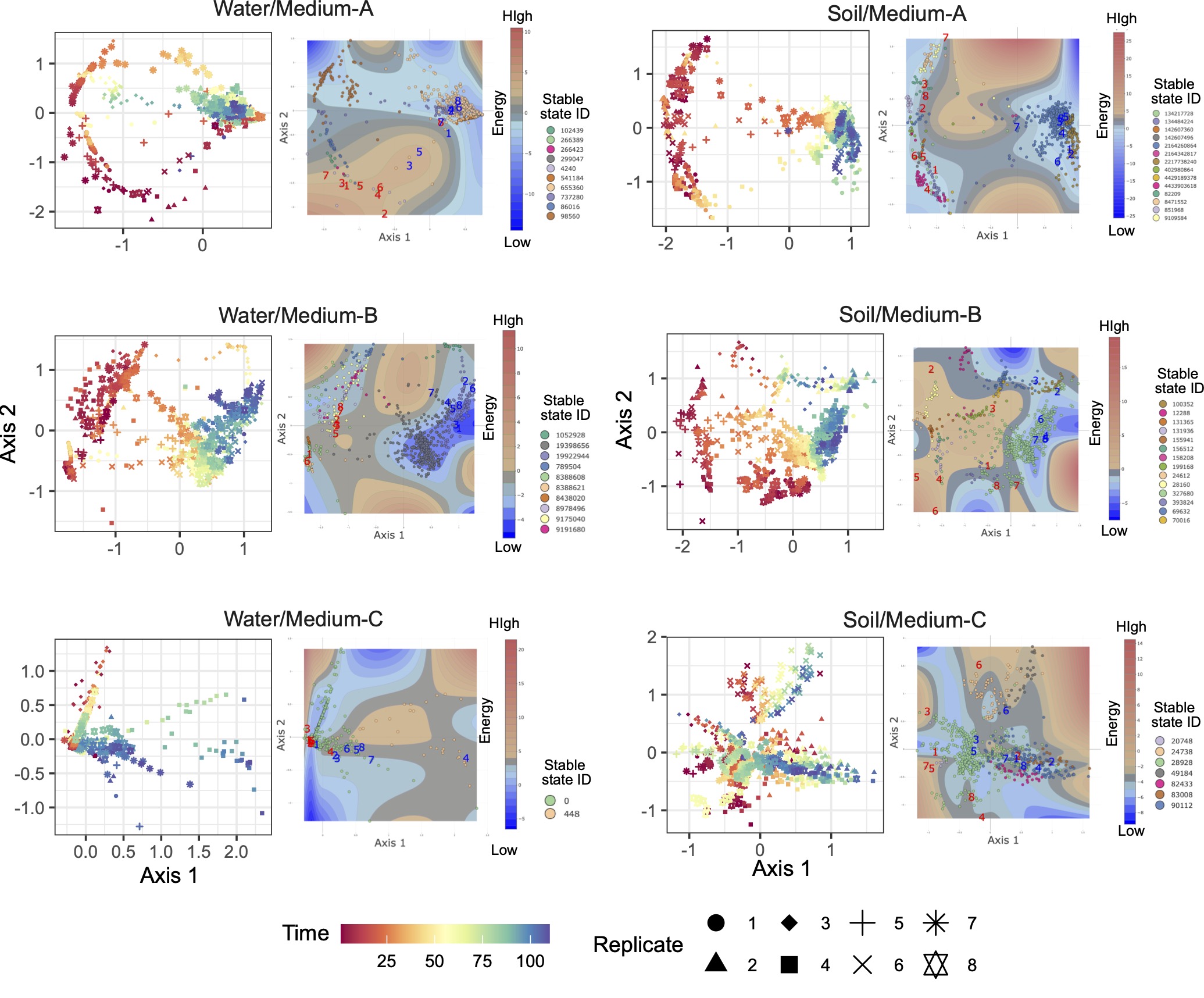
**

**Additional file 5: Fig. S5** Distribution of stable states on the energy landscapes. The community structure of respective time points on NMDS axes (left) and reconstructed energy landscape on the NMDS surface (right) are shown for each experimental treatment. Community states (ASV compositions) located at lower-energy regions are inferred to be more stable on the energy landscapes. On the energy landscape of each experimental treatment, community states (data points) belonging to the basin of the same stable states are indicated with the same colors. The shapes of the landscapes were inferred based on a smoothing spline method with optimized penalty parameters. Within the energy landscape, community states of Day 1 and Day 110 are respectively shown in red and blue numbers representing replicate communities.
