# Supplementary material for: Alternative stable states, nonlinear behavior, and predictability of microbiome dynamics
Source: Microbiome. 2023 Mar 29;11:63. doi: 10.1186/s40168-023-01474-5 (PMC10052866; doi:10.1186/s40168-023-01474-5)
Supplement: Supplementary file 7 — Additional file 6: Figure S6. Transitions between alternative stable states on the energy landscapes. [file 40168_2023_1474_MOESM6_ESM.docx]

**
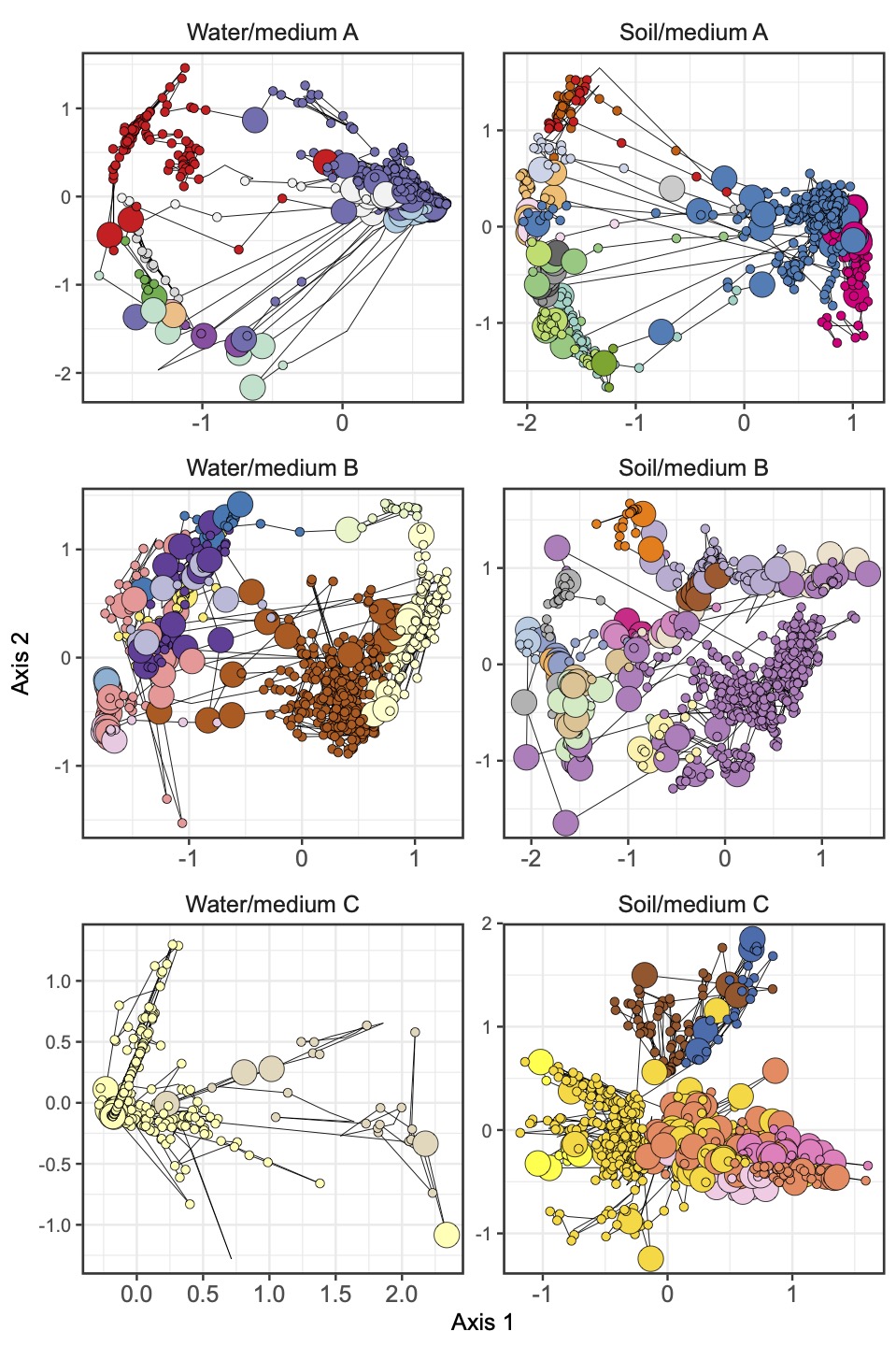
**

**Additional file 6: Fig. S6 Transitions between alternative** stable states on the energy landscapes. The community structure of respective stable states on NMDS axes is shown for each experimental treatment: the ordination of the data points is the same as Fig. S5. On the NMDS plot of each experimental treatment, community states (data points) belonging to the basin of the same stable states are indicated with the same colors. The time points toward which transitions between basins of stable states occurred are enlarged. The lines shows community transition from Day1 to Day 110.
