# Supplementary material for: Alternative stable states, nonlinear behavior, and predictability of microbiome dynamics
Source: Microbiome. 2023 Mar 29;11:63. doi: 10.1186/s40168-023-01474-5 (PMC10052866; doi:10.1186/s40168-023-01474-5)
Supplement: Supplementary file 8 — Additional file 7: Figure S7. Histogram of optimal embedding dimension (E) of ASVs. [file 40168_2023_1474_MOESM7_ESM.docx]

**
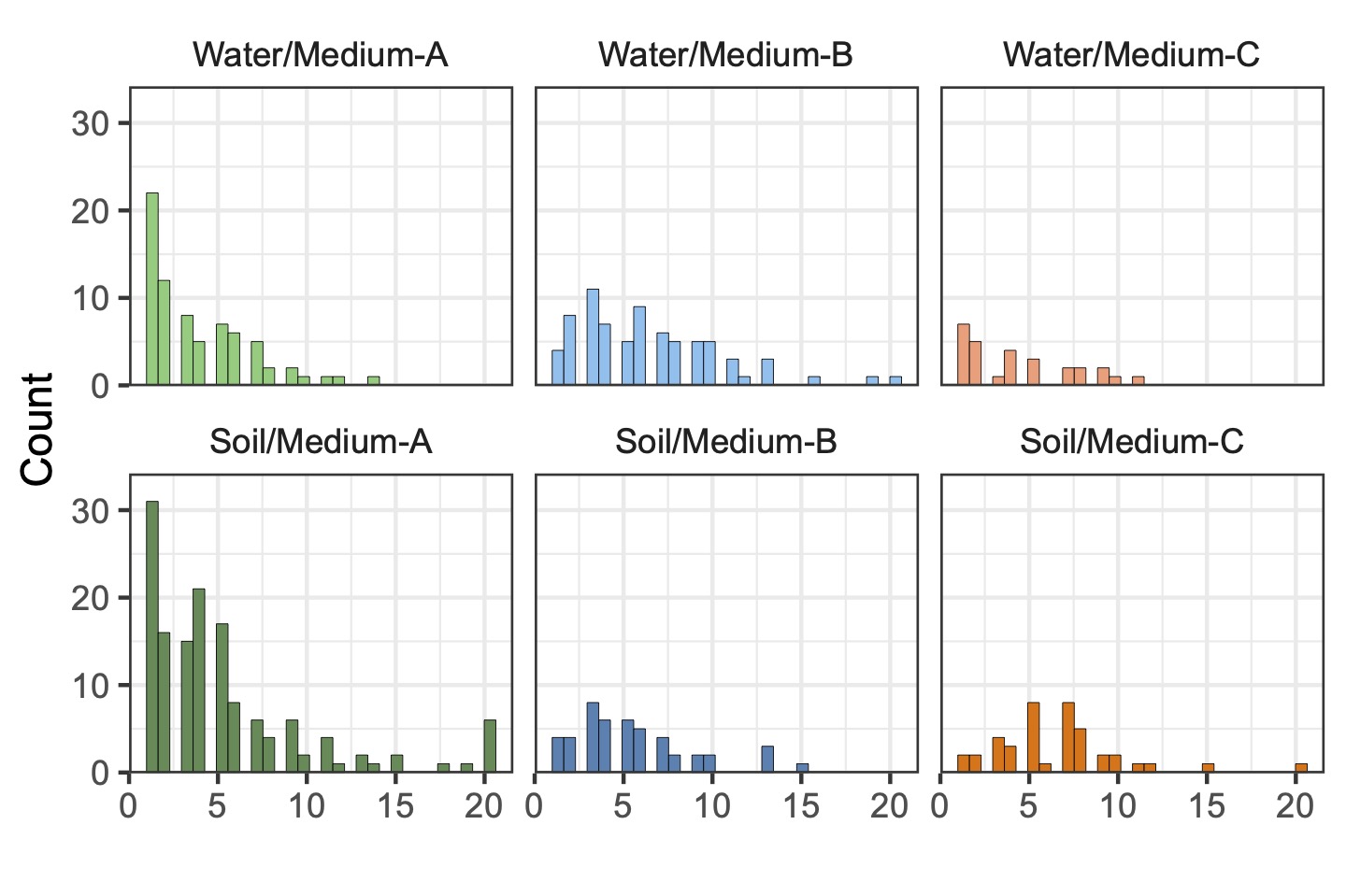
**

**Additional file 7: Fig. S7** Histogram of optimal embedding dimension (*E*) of ASVs. Optimal embedding dimensions were explored for respective ASVs based on simplex projection within the range from 1 to 20.
