# Supplementary material for: Alternative stable states, nonlinear behavior, and predictability of microbiome dynamics
Source: Microbiome. 2023 Mar 29;11:63. doi: 10.1186/s40168-023-01474-5 (PMC10052866; doi:10.1186/s40168-023-01474-5)
Supplement: Supplementary file 9 — Additional file 8: Figure S8. Examples of population-level forecasting results. [file 40168_2023_1474_MOESM8_ESM.docx]

**
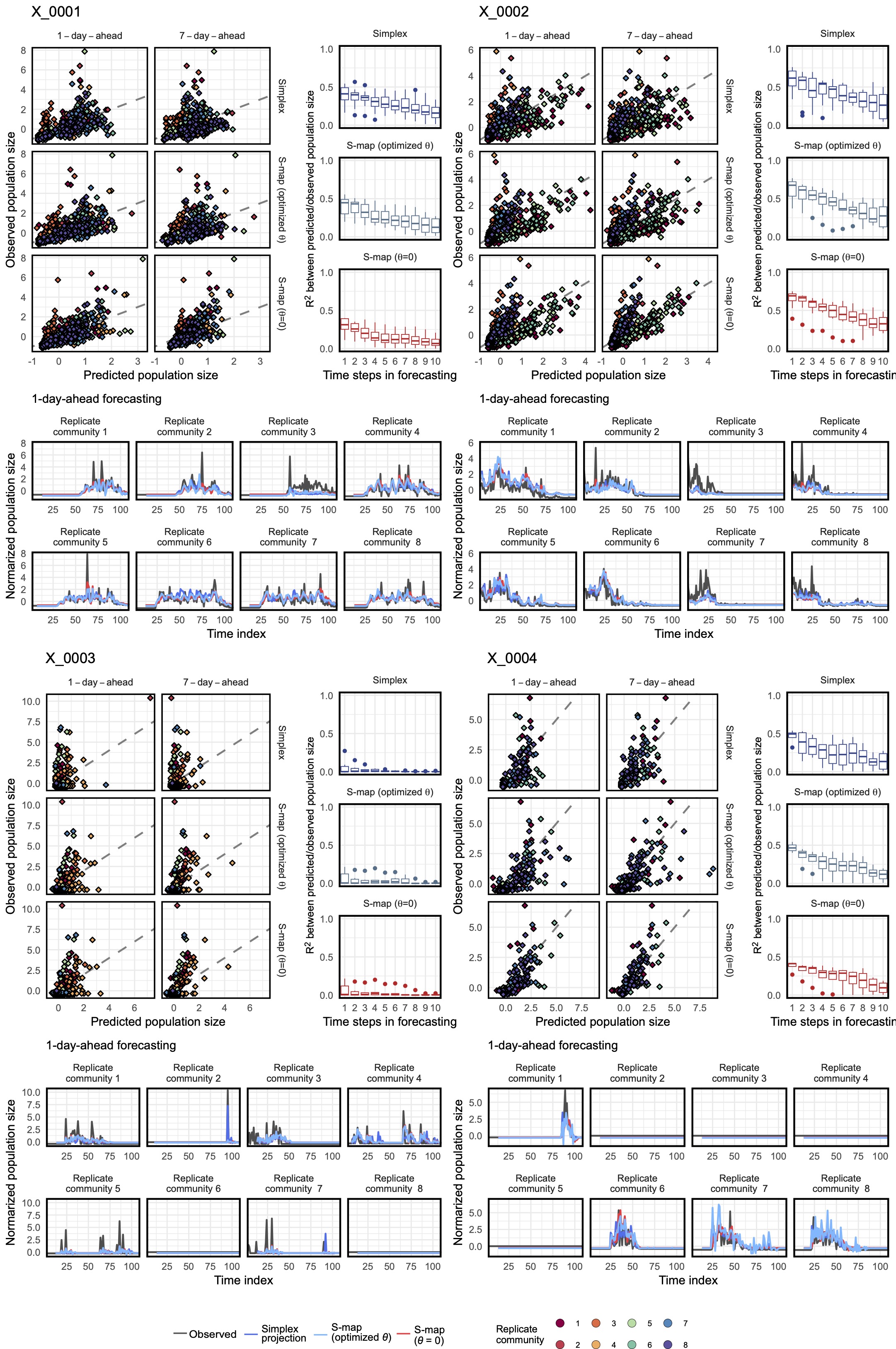
**

**Additional file 8: Fig. S8** Examples of population-level forecasting results. For each microbial ASV in each experimental treatment, correlations between predicted and observed abundance through the time-series (one-day-ahead and seven-day-ahead forecasting; top left), decay of R^2^ values between predicted and observed abundance (top right), and details of the time-series are shown. The prediction was based on simplex projection, S-map with optimized nonlinearity parameter (optimized *θ*), and S-map assuming linearity (*θ* = 0). For each target replicate community, the remaining seven replicate communities were used as references. Due to the large number of ASVs in the dataset, four ASVs in Water/Medium-B treatment are shown here as examples: the full results are available at the figshare repository (10.6084/m9.figshare.20653440).
