# Supplementary material for: Alternative stable states, nonlinear behavior, and predictability of microbiome dynamics
Source: Microbiome. 2023 Mar 29;11:63. doi: 10.1186/s40168-023-01474-5 (PMC10052866; doi:10.1186/s40168-023-01474-5)
Supplement: Supplementary file 10 — Additional file 9: Figure S9. Dependence of population-level forecasting results on reference database size. [file 40168_2023_1474_MOESM9_ESM.docx]

**
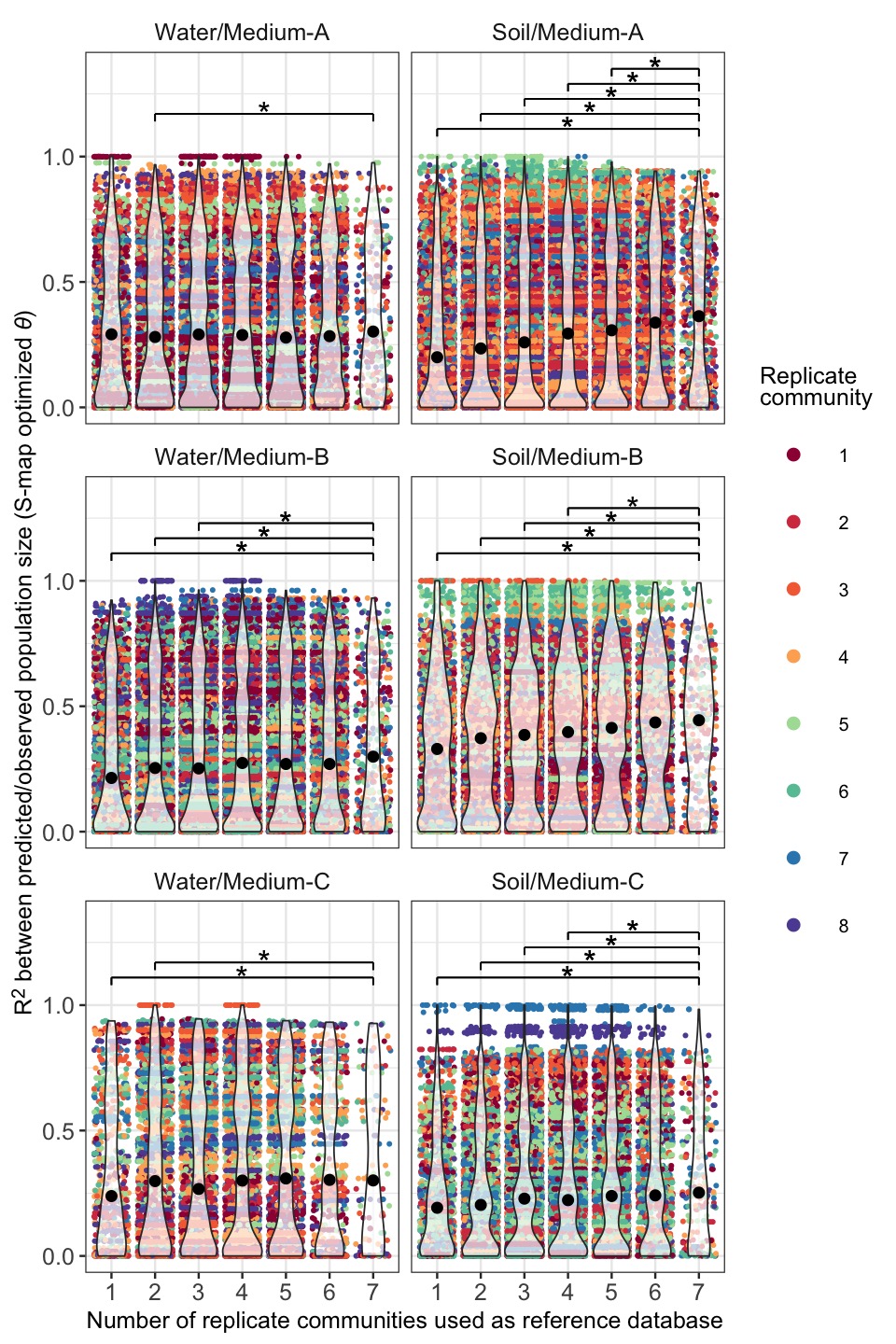
**

**Additional file 9: Fig. S9** Dependence of population-level forecasting results on reference database size. The population size of each microbial ASV in a target replicate community was forecasted with S-map (optimized *θ*) based on reference databases (Fig. 3a). The forecasting was performed for each number of reference databases defined on the horizontal axis. R^2^ values between predicted and observed population size (Fig. 3c) were calculated for each microbial ASV in each replicate community. An asterisk represents significant differences in forecasting skill (forecasting performance) between different numbers of reference databases in each experimental replicate: i.e., false discovery rate (FDR) based on Welch’s *t*-tests.
