# Supplementary material for: Alternative stable states, nonlinear behavior, and predictability of microbiome dynamics
Source: Microbiome. 2023 Mar 29;11:63. doi: 10.1186/s40168-023-01474-5 (PMC10052866; doi:10.1186/s40168-023-01474-5)
Supplement: Supplementary file 11 — Additional file 10: Figure S10. Comparison of predicted and observed community structure (seven-day-ahead forecasting). [file 40168_2023_1474_MOESM10_ESM.docx]

**
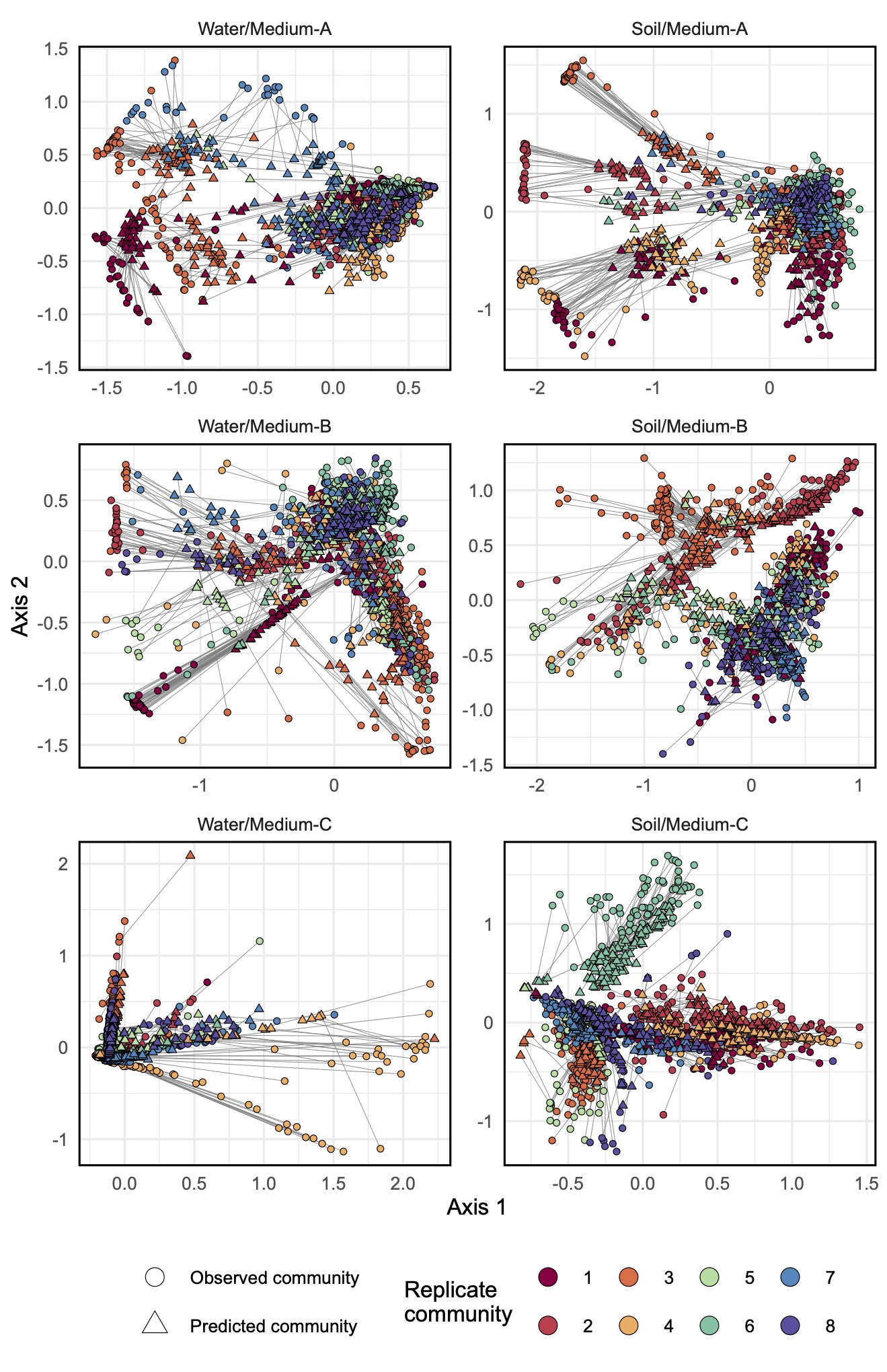
**

**Additional file 10: Fig. S10** Comparison of predicted and observed community structure (seven-day-ahead forecasting). By compiling the forecasting results of respective ASVs (Fig. 3; Additional file 8: Fig. S8), community compositions are predicted through the time-series. Predicted and observed community structure is linked for each day on the axes of NMDS (prediction based on S-map with optimized *θ*; one-day-ahead forecasting).
