# Supplementary material for: Alternative stable states, nonlinear behavior, and predictability of microbiome dynamics
Source: Microbiome. 2023 Mar 29;11:63. doi: 10.1186/s40168-023-01474-5 (PMC10052866; doi:10.1186/s40168-023-01474-5)
Supplement: Supplementary file 12 — Additional file 11: Figure S11. Distribution of prediction error in the community-level forecasting. [file 40168_2023_1474_MOESM11_ESM.docx]

**
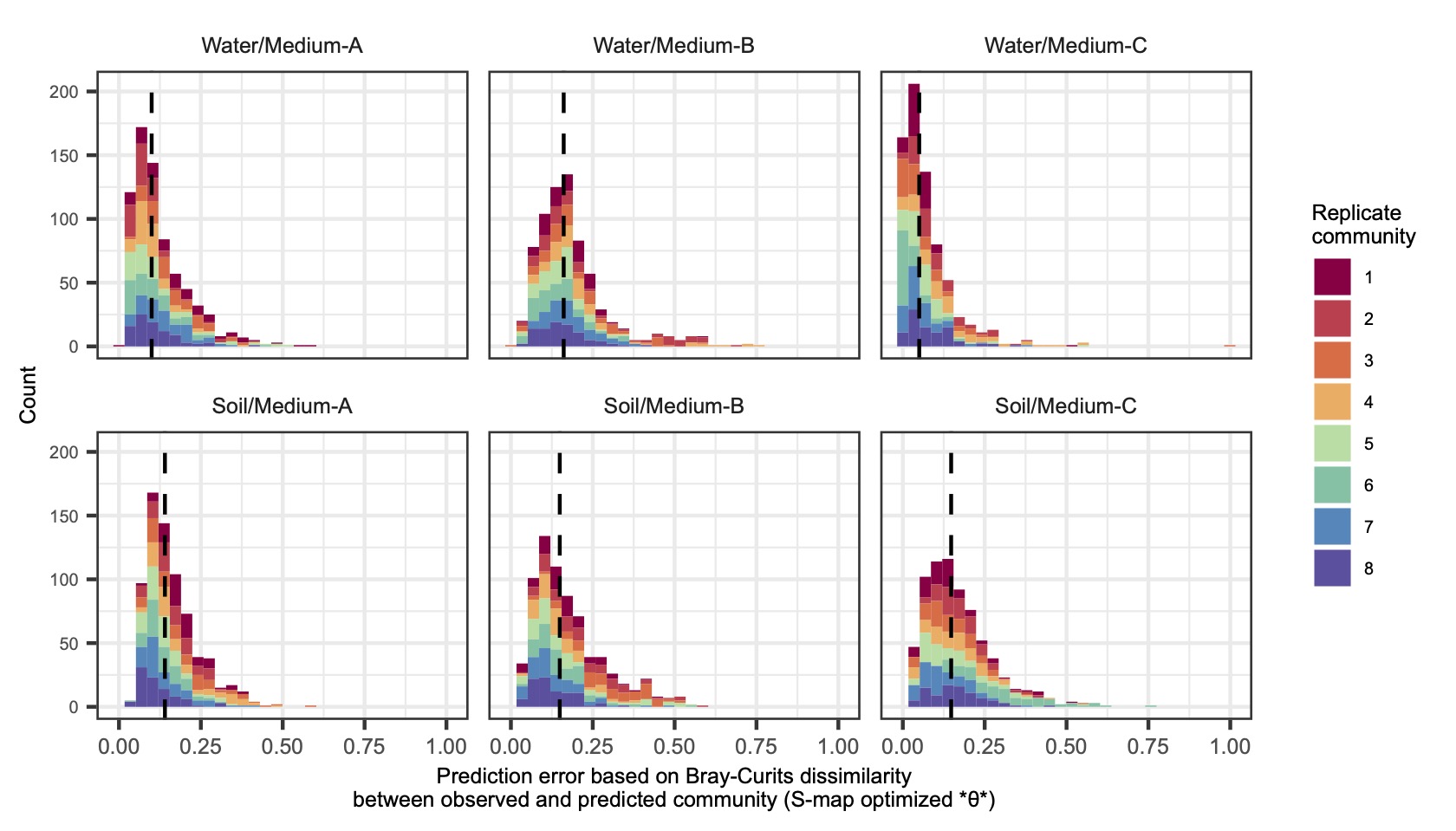
**

**Additional file 11: Fig. S11** Distribution of prediction error in the community-level forecasting. Bray-Curtis dissimilarity between predicted and observed community structure (Additional file 10: Fig. S10) was calculated as prediction error for each experimental treatment. Dashed lines represent median values.
