# Supplementary material for: Alternative stable states, nonlinear behavior, and predictability of microbiome dynamics
Source: Microbiome. 2023 Mar 29;11:63. doi: 10.1186/s40168-023-01474-5 (PMC10052866; doi:10.1186/s40168-023-01474-5)
Supplement: Supplementary file 13 — Additional file 12: Figure S12. Comparison of nonlinear and linear forecasting approaches. [file 40168_2023_1474_MOESM12_ESM.docx]

**
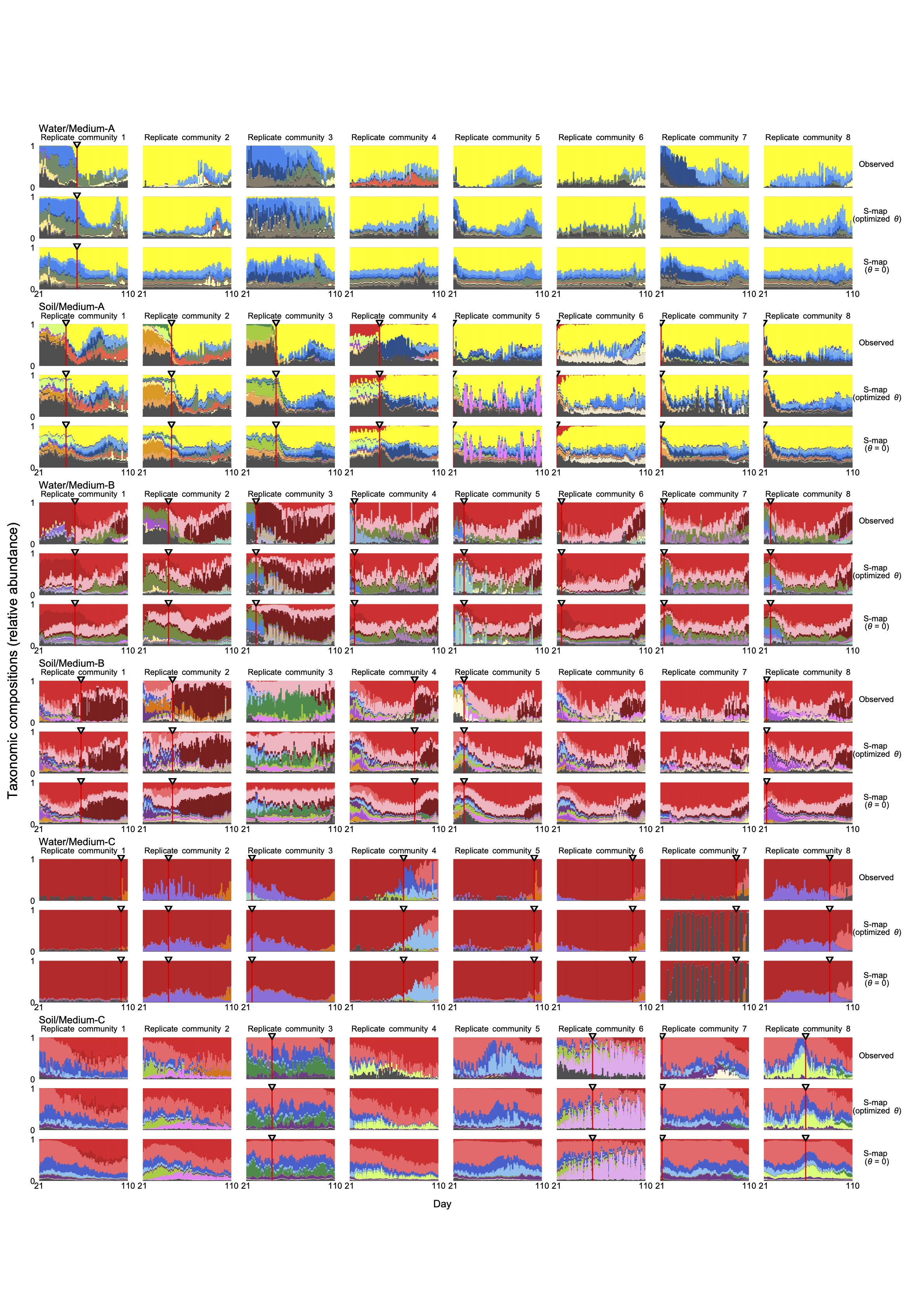
**

**Additional file 12: Fig. S12** Comparison of nonlinear and linear forecasting approaches. Throughout the time-series, S-map nonlinear forecasting results are shown with observed community compositions and linear forecasting results (seven-day-ahead prediction). For the direct comparison of nonlinear and linear forecasting methods, S-map results with optimized nonlinearity parameter were compared with results of S-map assuming linear dynamics for all ASVs (*θ* = 0). Note that forecasting is inapplicable to the beginning of the time-series depending on embedding dimensions and forecasting time steps. A vertical line represents the timing of the greatest community compositional change in each replicate community.
