# Supplementary material for: Alternative stable states, nonlinear behavior, and predictability of microbiome dynamics
Source: Microbiome. 2023 Mar 29;11:63. doi: 10.1186/s40168-023-01474-5 (PMC10052866; doi:10.1186/s40168-023-01474-5)
Supplement: Supplementary file 14 — Additional file 13: Figure S13. Candidates of signal indices for anticipating abrupt community changes. [file 40168_2023_1474_MOESM13_ESM.docx]

**
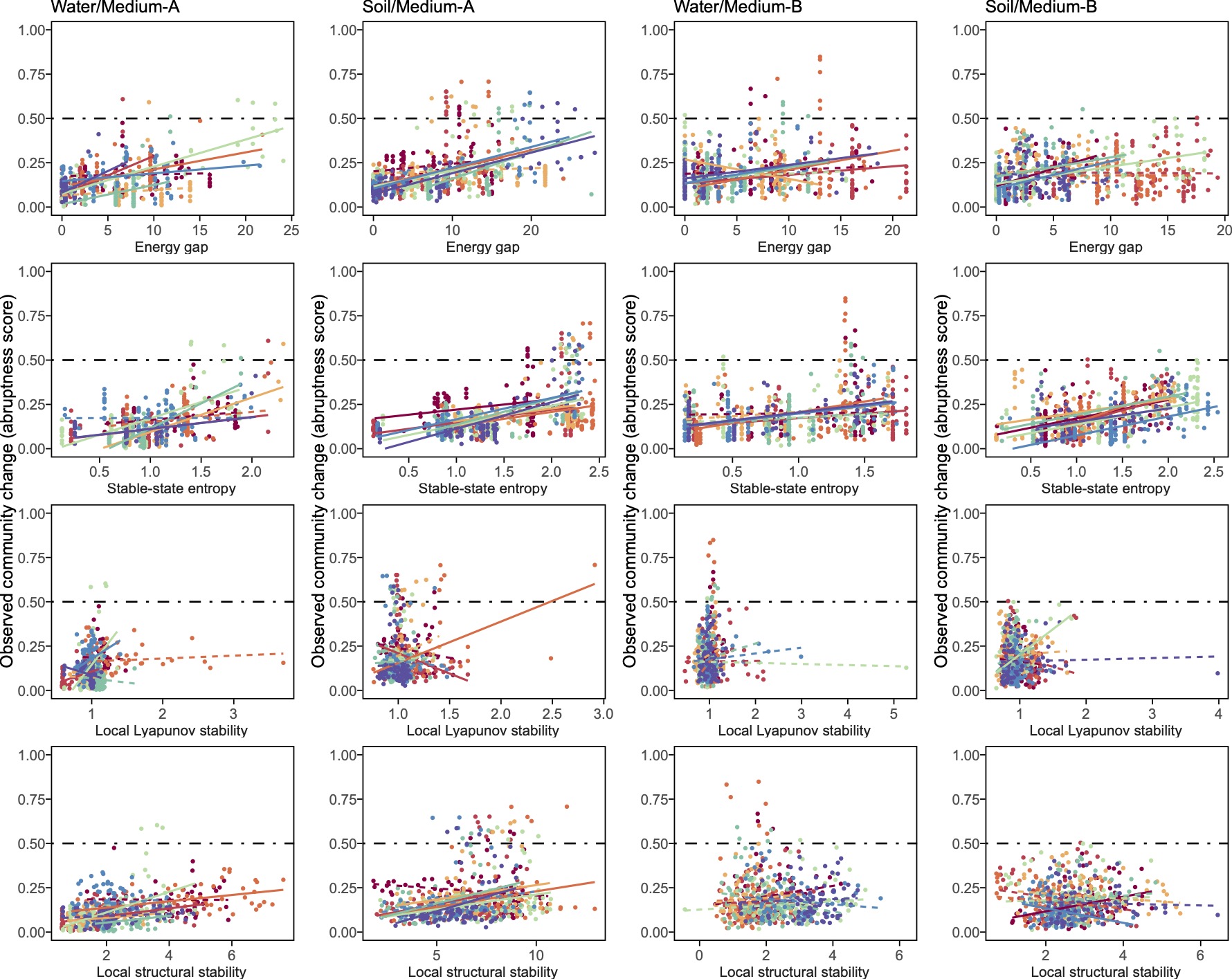
**

**Additional file 13: Fig. S13** Candidates of signal indices for anticipating abrupt community changes. Relationships between signal index values and observed community-compositional changes are shown for seven-day-ahead forecasting. For each index of potential early-warning signals, association with the degree of community-compositional changes (abruptness scores) was examined for each time lag between signal indices and observed abruptness. The indices examined were the energy gap and stable-state entropy of the energy landscape analysis and the local Lyapunov stability and local structural stability of empirical dynamic modeling. Significant/non-significant regressions within respective replicates are shown with solid/dashed lines for each panel.
