# Supplementary material for: Alternative stable states, nonlinear behavior, and predictability of microbiome dynamics
Source: Microbiome. 2023 Mar 29;11:63. doi: 10.1186/s40168-023-01474-5 (PMC10052866; doi:10.1186/s40168-023-01474-5)
Supplement: Supplementary file 15 — Additional file 14: Figure S14. Dependence of relationship between signal index values and observed community-compositional changes on time steps in forecasting. [file 40168_2023_1474_MOESM14_ESM.docx]

**
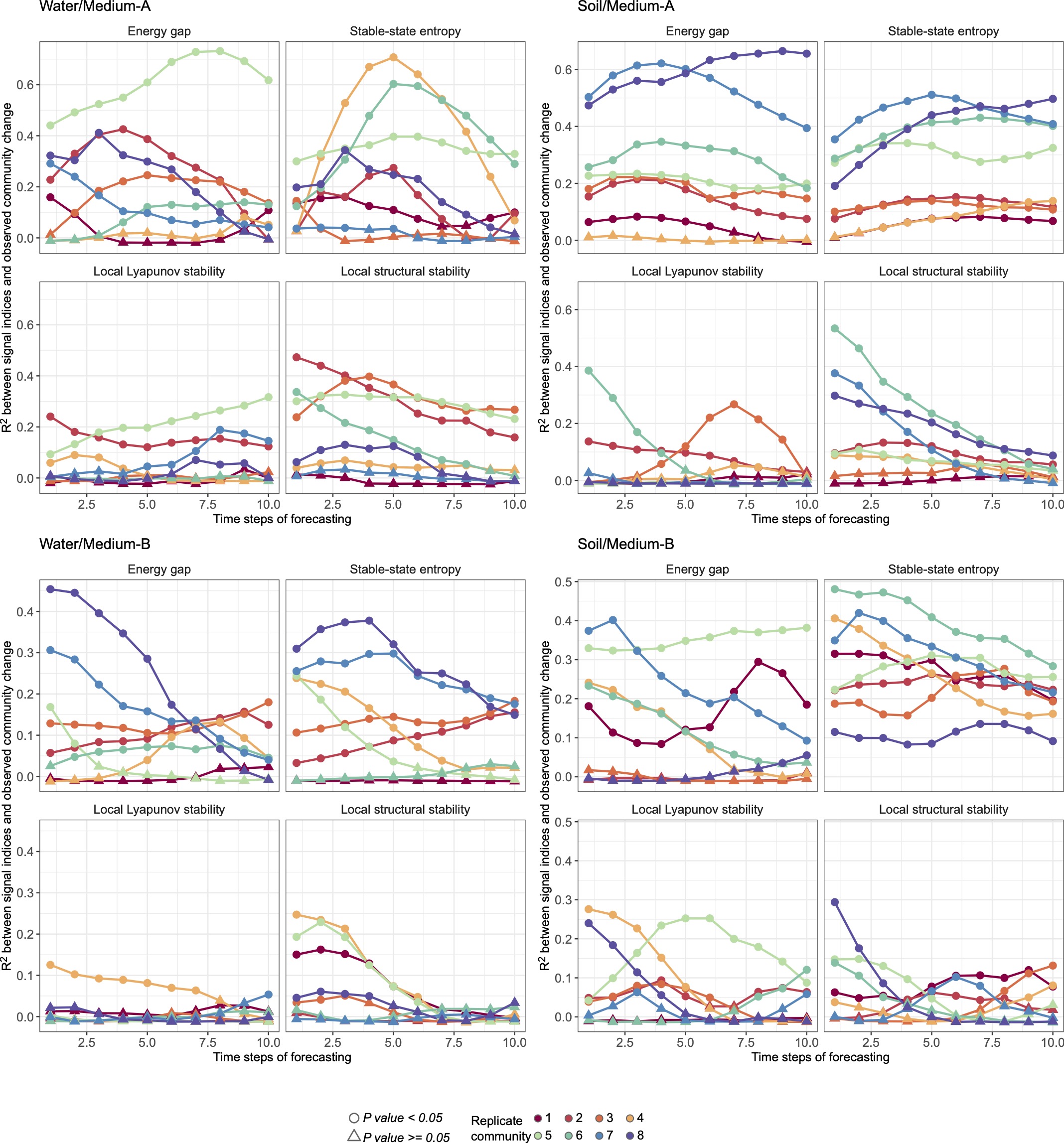
**

**Additional file 14: Fig. S14** Dependence of relationship between signal index values and observed community-compositional changes on time steps in forecasting. R^2^ values between signal index values and community-change abruptness (Additional file 13: Fig. S13) were calculated for each time step in forecasting within the range from 1 to 10.
