# Supplementary material for: Alternative stable states, nonlinear behavior, and predictability of microbiome dynamics
Source: Microbiome. 2023 Mar 29;11:63. doi: 10.1186/s40168-023-01474-5 (PMC10052866; doi:10.1186/s40168-023-01474-5)
Supplement: Supplementary file 16 — Additional file 15: Figure S15. ROC analysis of diagnostic performance. [file 40168_2023_1474_MOESM15_ESM.docx]

**
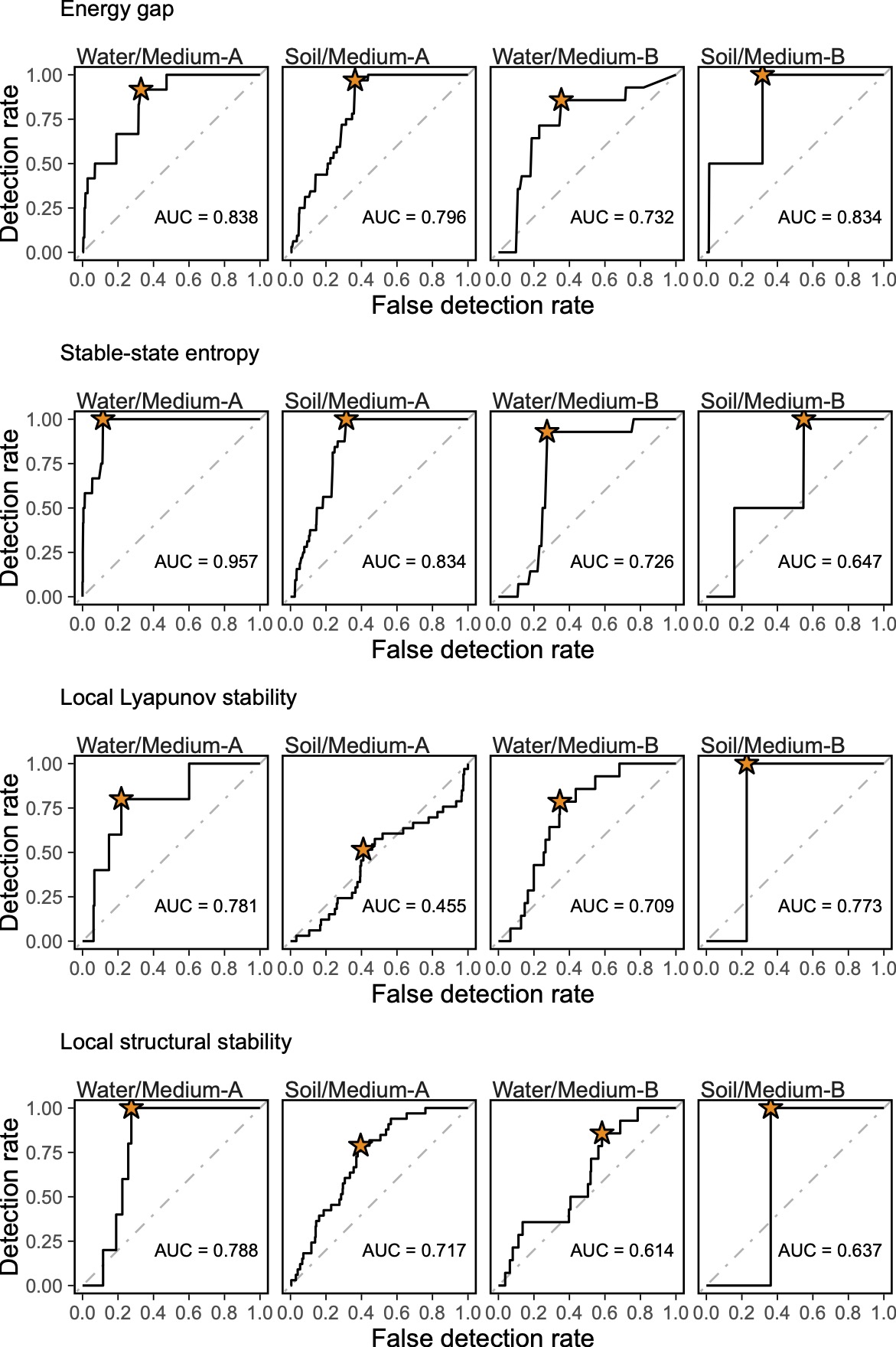
**

**Additional file 15: Fig. S15** ROC analysis of diagnostic performance. On the two-dimensional surface of detection- and false-detection rates of abrupt community changes (abruptness > 0.5), area under the curve (AUC) and optimal detection rate (asterisk) were calculated (top panels) for local structural stability or energy gap. Optimal diagnostic threshold of local structural stability or energy gap for warning abrupt community changes was then obtained for each treatment based on the Youden index (bottom panels). Note that abrupt community changes were absent in Medium-C treatments and that the threshold for Soil/Medium-B treatment was unreliable due to the small number of time points with abruptness > 0.5 (Additional files 4 and 13: Figs. S4 and S13).
